# Impact of antiphospholipid antibodies on cardiac valve lesions in systemic lupus erythematosus: a systematic review and meta-analysis

**DOI:** 10.1007/s10238-024-01406-z

**Published:** 2024-07-03

**Authors:** Siyun Chen, Yangzhong Zhou, Chuhan Wang, Hui Jiang, Yuan Zhao, Jiuliang Zhao, Can Huang, Mengtao Li, Yan Zhao

**Affiliations:** 1 Department of Rheumatology and Clinical Immunology, Peking Union Medical College Hospital, Chinese Academy of Medical Sciences, Peking Union Medical College, Beijing, 100730 China; 2 National Clinical Research Center for Dermatologic and Immunologic Diseases (NCRC-DID), Ministry of Science and Technology, Beijing, 100730 China; 3 State Key Laboratory of Complex Severe and Rare Diseases, Peking Union Medical College Hospital, Beijing, 100730 China; 4 Key Laboratory of Rheumatology and Clinical Immunology, Ministry of Education, Beijing, 100730 China

**Keywords:** Systemic lupus erythematosus, Antiphospholipid antibodies, Heart valve disease, Meta-analysis

## Abstract

This meta-analysis assesses antiphospholipid antibodies’ (aPLs) impact on heart valve disease in Systemic Lupus Erythematosus (SLE) patients. We searched PubMed, Embase, Cochrane, and Web of Science up to January 2024 for comparative studies of heart valve disease in aPL-positive versus aPL-negative SLE patients. Fixed-effect or random-effect models were used to synthesize data, with *I*^2^ and sensitivity analyses for heterogeneity and the trim-and-fill method for publication bias. Including 25 studies with 8089 patients, of which 919 had valvular changes, aPLs significantly increased the risk of heart valve disease (OR = 2.24, 95% CI: 1.58–3.18, *p* < 0.001). Lupus anticoagulant (LA) indicated the highest risk (OR = 4.90, 95% CI: 2.26–10.60, *p* < 0.001), anticardiolipin antibodies (aCL) doubled the risk (OR = 2.69, 95% CI: 1.47–4.93, *p* = 0.001), and anti-β2 glycoprotein I (aβ2GPI) showed a 70% increase (OR = 1.70, 95% CI: 1.17–2.45, *p* = 0.005). Valve-specific analysis indicated the mitral valve was most commonly involved (26.89%), with higher occurrences in aPL-positive patients (33.34% vs. 15.92%, *p* = 0.053). Aortic and tricuspid valve involvements were 13.11% vs. 5.42% (*p* = 0.147) and 12.03% vs. 8.52% (*p* = 0.039), respectively. Pulmonary valve disease was rare and similar across groups (1.01% in aPL-positive vs. 1.52% in aPL-negative). Significantly, only tricuspid valve disease showed increased risk in aPL-positive patients (OR = 2.66, 95% CI: 1.05–6.75, *p* = 0.039). APLs notably increase the risk of heart valve disease in SLE patients, with a pronounced effect on tricuspid valve involvement. Regular cardiac assessments for aPL-positive SLE patients are crucial for timely intervention and improved prognosis.

**Supplementary Information:**

The online version contains supplementary material available at 10.1007/s10238-024-01406-z.

## Introduction

Systemic Lupus Erythematosus (SLE) is a complex autoimmune disorder characterized by diffuse connective tissue inflammation. It is distinguished by a spectrum of autoantibodies, predominantly antinuclear antibodies, leading to multi-systemic involvements [1]. Cardiac manifestations in SLE are common, occurring in over half of the patients. Heart valve disease, a significant aspect of these manifestations [2], exhibits a prevalence ranging from 10 to 75% [3–5]. This involvement typically presents with morphological alterations like valve thickening and vegetations, alongside functional impairments such as regurgitation and stenosis. While a majority of patients remain asymptomatic, approximately 20% may develop clinical symptoms, including systemic embolic events (e.g., stroke, peripheral arterial embolism, intestinal ischemia), dyspnea, and heart failure, with 1–4% eventually necessitating surgical intervention due to severe heart failure or recurrent embolic episodes. The pathophysiological mechanisms underlying valvular damage in these patients are complex and not yet fully understood. Based on clinical analyses and pathological observations, they likely result from multiple factors, including platelet aggregation and thrombus formation induced by pathogenic antibodies, such as antiphospholipid antibodies (aPLs), as well as immune complex-mediated inflammatory infiltration [2, 6–9].

APLs are autoantibodies targeting phospholipids or their binding proteins. The most extensively studied antibodies, which are included in the classification criteria for antiphopholipid syndrome (APS), are anticardiolipin antibodies (aCL), anti-β2 glycoprotein I antibodies (aβ2GPI), and lupus anticoagulant (LA). Clinically significant, aPLs are implicated in thrombosis, adverse pregnancy outcomes, heart valve disease, and thrombocytopenia, and other systemic effects [10]. Approximately 30–45% of SLE patients are positive for aPLs [11, 12]. Literature suggesting a potential link between aPLs and an elevated risk of heart valve disease in SLE patients, although findings have been inconsistent [13, 14].

Given these disparities, this study aims to systematically review and perform a meta-analysis on the existing research concerning the association between heart valve disease and aPLs in SLE patients.

## Methods

### Search strategy

This systematic review and meta-analysis, registered on PROSPERO (CRD42023492344) and adhering to PRISMA guidelines, involved a comprehensive literature search across PubMed, Embase, Cochrane, and Web of Science databases. The search, spanning from the inception of each database to January 2024, focused on case–control, cross-sectional, and cohort studies examining the impact of aPLs on heart valves in SLE patients. Keywords and subject terms included variations of “Systemic Lupus Erythematosus,” “Antibodies, Antiphospholipid,” “Antibodies, Anticardiolipin,” and “Lupus Coagulation Inhibitor,” along with specific terms related to heart valves and Libman-Sacks disease. Studies in languages other than English and Chinese, and those for which full-text articles could not be retrieved were excluded. An additional article [15] was identified and included after reviewing the reference lists of retrieved papers. Detailed search terms are shown in Supplementary Table S1.

### Inclusion criteria and quality assessment

Studies were selected based on the following criteria: (1) study design as cohort, case–control, or cross-sectional; (2) diagnosis of SLE in subjects; (3) confirmation of heart valve disease via Transthoracic Echocardiography (TTE) or Transesophageal Echocardiography (TEE); and (4) availability of detailed patient data regarding heart valve disease and aPL status. Quality assessment employed the Newcastle–Ottawa Scale for cohort and case–control studies, and the Agency for Healthcare Research and Quality scale (AHRQ) for cross-sectional studies.

### Data extraction and analysis

Data extraction encompassed study characteristics such as author, publication year, country, follow-up duration, patient demographics, aPL testing parameters, heart valve involvement, and additional clinical details. Statistical analysis was performed using R software version 4.3.1. Fixed-effect or random-effect models were used to compare aPL positivity rates in SLE patients with and without heart valve disease. Effect size was estimated using odds ratios (ORs) and 95% confidence intervals (CIs). Heterogeneity was quantified using the *Q* test and *I*^2^ test, categorized as low (0–25%), moderate (26–50%), substantial (51–75%), or considerable (> 75%) heterogeneity [16]. Sources of heterogeneity were explored through Baujat plots, Galbraith radial plots, and sensitivity analysis. Publication bias was assessed using Egger’s test, the trim-and-fill method, and enhanced funnel plots, with bias inferred from Egger’s test, *p* < 0.05, or discrepancies in the trim-and-fill method. The robustness of findings was evaluated via sensitivity analysis employing the leave-one-out approach.

## Results

### Article selection and study population characteristics

**Fig. 1:**
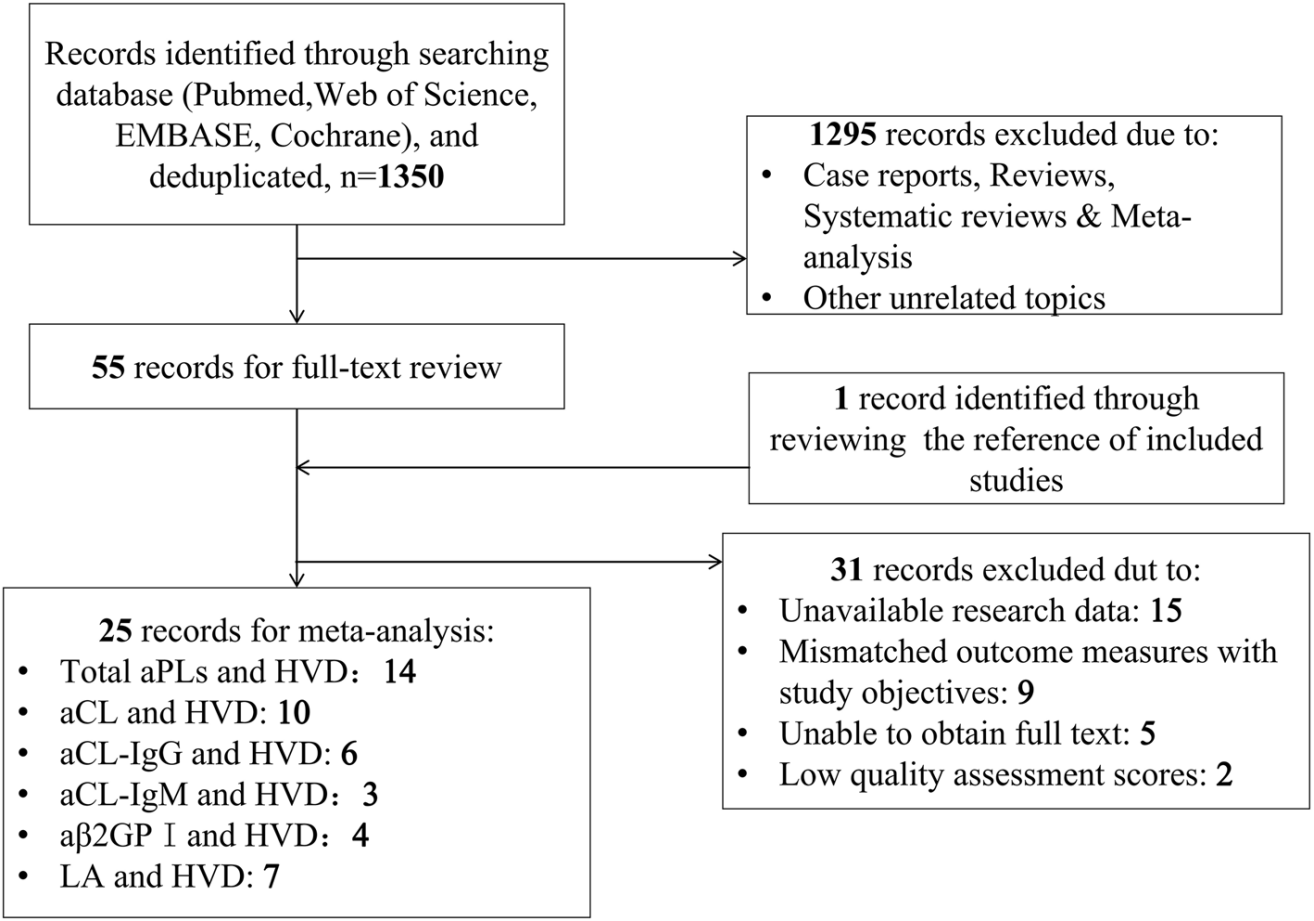
Flowchart of study selection

Our initial database search yielded 1839 articles. Following deduplication, 1350 articles were screened for relevance. This scrutiny resulted in 56 articles eligible for full-text review. Of these, 15 articles were excluded because, although they contained data of aPLs or valvular lesions, they lacked specific information on the distribution and association between the two. Another nine articles were excluded because their grouping methodologies of heart valve diseases were not in alignment with our study objectives, and five were inaccessible in full-text. Two articles were excluded from the study due to low quality assessment scores. This selection process in the inclusion of 25 articles for detailed analysis. The methodical progression of our search and selection is delineated in flowchart (Fig. 1), and the quality of each included article is systematically appraised in Table 1 and Supplementary Tables S2 and S3.

**Table 1:** Bias assessment of included studies

Author	Year	Selection	Comparability	Exposure	Outcome
Cohort study					
Nihoyannopoulos et al. [17]	1990	2	1	–	3
Khamashta et al. [18]	1990	3	2	–	2
Sturfelt et al. [19]	1992	3	2	–	3
Cervera et al. [20]	1992	3	2	–	2
Jouhikainen et al. [21]	1994	3	2	–	3
Moyssakis et al. [3]	2007	3	2	–	2
Kampolis et al. [22]	2014	3	2	–	3
Case–control study					
Ruid et al. [14]	2018	2	2	2	–
Yoo et al. [13]	2020	3	2	3	–
He et al. [23]	2022	3	2	3	–
Cross–sectional study					
Roldan et al. [24]	1992	8			
Meyer et al. [25]	1995	9			
Gabrielli et al. [26]	1995	8			
Bengtsson et al. [27]	1996	6			
Falcao et al. [28]	2002	9			
Leszczynski et al. [29]	2003	8			
Shahin et al. [30]	2004	8			
Roldan et al. [31]	2005	7			
Farzaneh–Far et al. [32]	2006	9			
Roldan et al. [33]	2007	7			
Tarr et al. [34]	2007	7			
Heredia et al. [35]	2014	7			
Taraborelli et al. [15]	2016	8			
Watad et al. [36]	2017	6			
Liao et al. [37]	2022	8			

Quality assessment employed the Newcastle–Ottawa Scale for cohort and case–control studies, and the Agency for Healthcare Research and Quality scale (AHRQ) for cross-sectional studies

The meta-analysis encompassed data from 8089 patients with SLE as reported in these 25 selected studies, with a total of 919 exhibiting valvular changes. Comprehensive demographic information, disease duration, patterns of organ involvement, disease activity indices, and treatment protocols for these patients are collected and presented in Table 2.

**Table 2:** Baseline characteristics of SLE patients included in the study

First author–year of publication	Age (HVD + vs. HVD−)	Gender F–M	Disease duration	SLEDAI	Organ involvement	Treatment
Liao et al. (2022)	35 ± 8 (36 ± 10 vs. 33 ± 8)	89/15	8.4 ± 6.2 vs. 5.5 ± 5.6 years	–	–	–
He et al. (2022)	32 ± 9 vs. 32 ± 9	72/8	21.0 (2.4–73.5) vs. 24.9 (2.4–114.3) months	10.0 (5.5–13.8) vs. 12.0 (8.0–16.0)	Raynaud’s phenomenon: 2/20 vs. 7/60; Photosensitivity: 2/20 vs. 7/60; Hair loss: 4/20 vs. 24/60; Oral ulcers: 2/20 vs. 6/60; Proteinuria: 13/20 vs. 40/60; Renal insufficiency: 3/20 vs. 11/60; Cognitive dysfunction: 3/20 vs. 10/60; Hemolytic anemia: 3/20 vs. 3/60; Leukopenia: 8/20 vs. 17/60; Thrombocytopenia: 11/20 vs. 20/60	Antithrombotic: 20/20
Yoo et al. (2020)	28.4 (24.9 (22.9–39.5) vs. 28.9 (24.1–39.0))	40/0	2.5 (0–124.0) vs. 0 (0–2.1) months	10 (8–14) vs. 12 (9–21)	Cutaneous: 17/40 (4/11 vs. 13/29); Arthritis: 16/40 (4/11 vs. 12/29); Serositis: 18/40 (3/11 vs. 15/29); Renal: 22/40 (7/11 vs. 15/29); Neurological: 8/40 (3/11 vs. 5/29); Hemolytic anemia: 7/40 (1/11 vs. 6/29); Leucopenia: 28/40 (6/11 vs. 22/29); Thrombocytopenia: 15/40 (5/11 vs. 10/29)	Antithrombotic: 7/11 (HVD); High dose GC: 5/11; Immunosuppressants: 6/11;
Ruiz et al. (2018)	34 ± 16.7	65/5	6.8 ± 7.2 years	–	–	–
Watad et al. (2017)	50.2 ± 17.4	4113/905	–	–	–	–
Taraborelli et al. (2016)	31 ± 11	300/17	19 (5–53) years	12 ± 6	Raynaud’s phenomenon: 139/317; Photosensitivity: 150/317; Hair loss: 84/317; Oral ulcers: 113/317, Acute cutaneous lupus: 176/317; Subacute cutaneous lupus: 17/317; Chronic cutaneous lupus: 20/317; Glomerulonephritis: 108/317; Epilepsy: 24/317; Psychosis: 12/317; Myelitis: 2/317; Mood disorders: 71/317; Cognitive dysfunction: 38/317; Peripheral neuropathy: 23/317; Hemolytic anemia: 23/317; Leukopenia: 88/317; Thrombocytopenia: 58/317	–
Kampolis et al. (2014)	–	–	–	–	–	–
Heradia et al. (2014)	50 ± 12	75/16	83 (12–156) months	–	–	–
Tarr et al. (2007)	34.3 ± 12.1	248/34	13.6 ± 8.5 years	5.8 (2–12)	Livedo reticularis: 29/272; Raynaud’s phenomenon: 112/272; Non-stroke CNS events: 99/272; Thrombocytopenia: 38/272; Pulmonary hypertension: 1/272	–
Moyssakis et al. (2007)	43.14 ± 15.10 vs. 38.73 ± 12.27	296/46	13.13 ± 11.26 vs. 5.91 ± 5.64 years	6.91 ± 3.18 vs. 3.82 ± 2.63 (ECLAM)	Renal disorders: 53/342; Hemolytic anemia: 55/342; Thrombocytopenia: 55/342	–
Roldan et al. (2007)	–	32/37	–	–	ESRD: 4/37; Anemia: 19/37; Leukopenia: 13/37; Thrombocytopenia: 21/37	–
Farzaneh-Far et al. (2006)	44.0 ± 13.7 vs. 2.8 ± 13.0	–	164 ± 108 vs. 138 ± 107 months	5.8 ± 5.6 vs. 3.7 ± 4.9 (aPLs + vs. aPLs−)	–	GC: 181/200; CTX: 42/200, AZA: 65/200; HCQ: 153/200
Roldan et al. (2005)	40 ± 12	23/28	11.4 ± 8.7 years	–	–	–
Shahin et al. (2004)	25.8 ± 9.5 (25.9 ± 8.8 vs. 25.8 ± 9.8)	–	3.9 ± 3.7 (4.1 ± 3.7 vs. 3.9 ± 3.8) years		Malar rash: 51/62 (15/19 vs. 36/43); Discoid rash: 5/62 (3/19 vs. 2/43); Photosensitivity: 45/62 (14/19 vs. 31/43); Oral ulcers: 36/62 (12/19 vs. 24/43); Arthritis: 26/62 (9/19 vs. 17/43); Renal disorders: 25/62(8/19 vs. 17/43); Neurological disorder: 35/62 (15/19 vs. 20/43); Serositis: 29/62 (10/19 vs. 19/43); Hematologic: 17/62 (5/19 vs. 12/43)	GC: 22.3 ± 11.3 (24.7 ± 11.9 vs. 21.3 ± 11.1) mg
Leszczyn ´ ski et al. (2003)	–	–	–	–	–	–
Falcao et al. (2002)	33.5 ± 10.6	67/3	5.8 ± 5.4 years	–	Cutaneous: 54/70; Renal: 51/70; NPSLE: 23/70; Leucopenia: 27/70; Thrombocytopenia: 11/70	GC: 65/70; Cytotoxins: 15/70
Bengtsson et al. (1996)					Malar rash: 35/78; Discoid rash: 21/78; Photosensitivity: 43/78; Oral ulcers: 10/78; Arthritis: 73/78; Renal disorders: 17/78; Neurological: 11/78; Hematologic: 38/78	–
Meyer et al. (1995)					Malar rash: 55/92; Photosensitivity: 33/92; Oral ulcers: 14/92; Arthritis: 79/92; Renal disorders: 39/92; Neurological: 24/92; Leucopenia: 48/92; Thrombocytopenia: 17/92	
Gabrielli et al. (1995)	–	–	–	–	–	–
Jouhikainen et al. (1994)	–	–	–	–	–	–
Cervera et al. (1992)	–	–	–	39 patients with active SLE	–	–
Sturfelt et al. (1992)	50 (20–48)	61/75	7 (1–39) years	–	–	–
Roldan et al. (1992)	37 ± 13 vs. 40 ± 10 (aPLs + vs. aPLs−)	49/54	9.3 ± 9.3 vs. 9.0 ± 7.3 years (aPLs + vs. aPLs−)	–	–	–
Nihoyannopoulos et al. (1990)	–	–	–	–	–	–
Khamashta et al. (1990)	–	–	–	–	–	–

Age and Disease Duration: These variables are reported as mean ± standard deviation (SD) or median and interquartile range (IQR) accordingly

The numbers in organ involvement and treatment column refer to the count of patients with organ involvement in the HVD + and HVD- groups, respectively

GC: glucocorticoid; CTX: cyclophosphamide; AZA: azathioprine; HCQ: hydroxychloroquine; SLEDAI: Systemic Lupus Erythematosus Disease Activity Index; ECLAM: European Consensus Lupus Activity Measurement; HVD: heart valve disease; aPLs: antiphospholipid antibodies; CNS: central nervous system; NPSLE: neuropsychiatric systemic lupus erythematosus; ESRD: end-stage renal disease

### Association between heart valve disease and aPLs in SLE patients

**Fig. 2:**
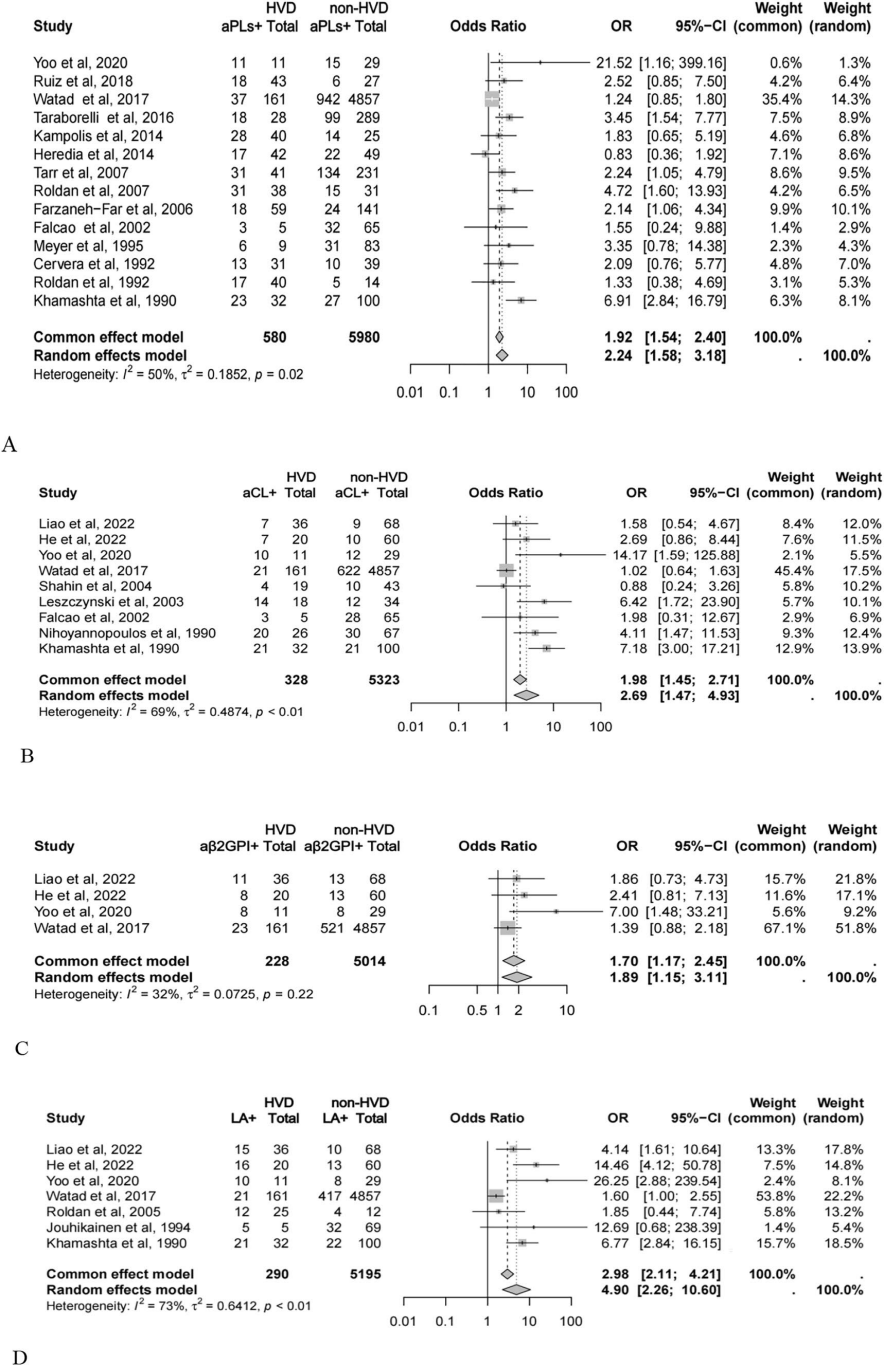
Risk of heart valve disease in SLE patients with different antiphospholipid antibodies

Among 6560 SLE patients assessed across 14 studies with aPLs results, 580 (8.87%) exhibited heart valve disease, with 269 (46.38%) being aPL-positive. In contrast, 1380 (23.08%) of the 5980 patients without heart valve disease were aPL-positive. Our meta-analysis demonstrated that aPL positivity approximately doubled the risk of heart valve disease in SLE patients (OR = 2.24, 95% CI: 1.58–3.18, *p* < 0.001). This analysis displayed moderate study heterogeneity (*I*^2^ = 50%, *p* = 0.018), underscoring a consistent association across studies (Fig. 2). Galbraith radial plots and Baujat plots pinpointed Watad A's and Kahamashta's studies as two primary sources of this heterogeneity, and with Watad A's contributing most to the result (Supplementary Figure S1A, B). Excluding each of these two studies separately from the analysis reduced the heterogeneity measured by *I*^2^ to be 32% and 31% (*p*>0.05), respectively. The ORs remained approximately consistent around 2 (2.43 and 1.76), as shown in Supplementary Fig. S2A.

In terms of aCL, analysis of 5651 patients from nine studies with available data revealed that 328 (5.80%) had heart valve disease, among whom 107 (32.62%) were aCL-positive. Among patients without heart valve disease, 14.34% were aCL-positive. The meta-analysis indicated that aCL positivity increased the risk of heart valve disease by nearly twofold (OR = 2.69, 95% CI: 1.47–4.93, *p* = 0.001), although with substantial heterogeneity (*I*^2^ = 69%, *p* = 0.001) (Fig. 2). Galbraith radial plots and Baujat plots pinpointed Watad A's and Kahamashta's studies as two primary sources of this heterogeneity, and with Watad A's contributing most to the result too (Supplementary Fig. S1C, D). A subsequent sensitivity analysis showed excluding these two studies from the analysis, respectively, markedly reduced heterogeneity (*I*^2^ = 41% and *I*^2^ = 58%) and reaffirmed the significant risk associated with aCL positivity (ORs were 3.43 and 1.63, respectively) (Supplementary Fig. S2B). And ORs of aCL-IgG and aCL-IgM were 5.06 (95% CI: 3.41–7.52) and 4.24 (95% CI: 2.40–7.49), from the analysis of 788 and 526 patients, respectively (Supplementary Fig. S3).

In studies involving aβ2GPI and LA, our analysis covered 5242 and 5485 patients, respectively. For the risk analysis of aβ2GPI positivity, 4.35% of patients had heart valve disease, with 21.93% being aβ2GPI-positive. This subgroup showed a 70% increased risk (OR = 1.70, 95% CI: 1.17–2.45, *p* = 0.005) with moderate heterogeneity (*I*^2^ = 32%, p = 0.222). Regarding the risk of LA positivity, 31.03% of the 290 patients with heart valve disease were LA-positive. Here, LA positivity was associated with an approximately fivefold increase in risk (OR = 4.90, 95% CI: 2.26–10.60, *p* < 0.001), yet with notable heterogeneity (*I*^2^ = 73%, *p* = 0.001). Further sensitivity analysis, particularly post-exclusion of Watad A's study, showed a substantial increase in risk, nearly sevenfold and reduction of *I*^2^ from 73 to 30%, still suggesting a strong link between LA positivity and heart valve disease in SLE patients (Supplementary Fig. S2C).

### Prevalence of different heart valve disease

**Fig. 3:**
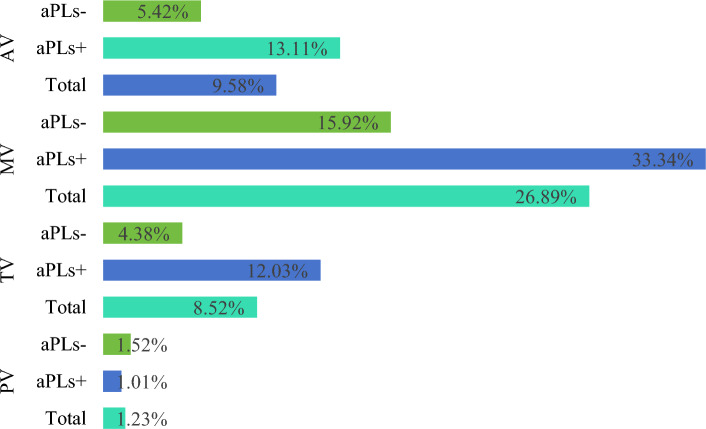
Prevalence and association of valvular diseases with antiphospholipid antibodies in SLE patients

We examined the prevalence of various heart valve diseases across four articles involving 244 SLE patients, with 99 exhibiting valvular changes. We found that the mitral valve was the most commonly involved (26.89%), followed by the aortic (9.58%), tricuspid (8.52%), and pulmonary valves (1.23%). Notably, aPL-positive patients exhibited an increased incidence of valve involvement compared to aPL-negative patients, with prevalence rates of 33.34% vs. 15.92% (*p* = 0.053) for the mitral valve, 13.11% vs. 5.42% (*p* = 0.147) for the aortic valve, and 12.03% vs. 4.38% (*p* = 0.039) for the tricuspid valve. Interestingly, pulmonary valve disease was slightly more prevalent in aPL-negative patients (1.52%) compared to aPL-positive patients (1.01%) (*p* = 0.791) (Fig. 3). However, our meta-analysis indicated that the presence of aPLs significantly increased the risk of tricuspid valve disease (OR = 2.66, 95%CI: 1.05–6.75), while the risk increases for the mitral, aortic, and pulmonary valves were not statistically significant (Supplementary Fig. S4).

### Cumulative meta-analyses

**Fig. 4:**
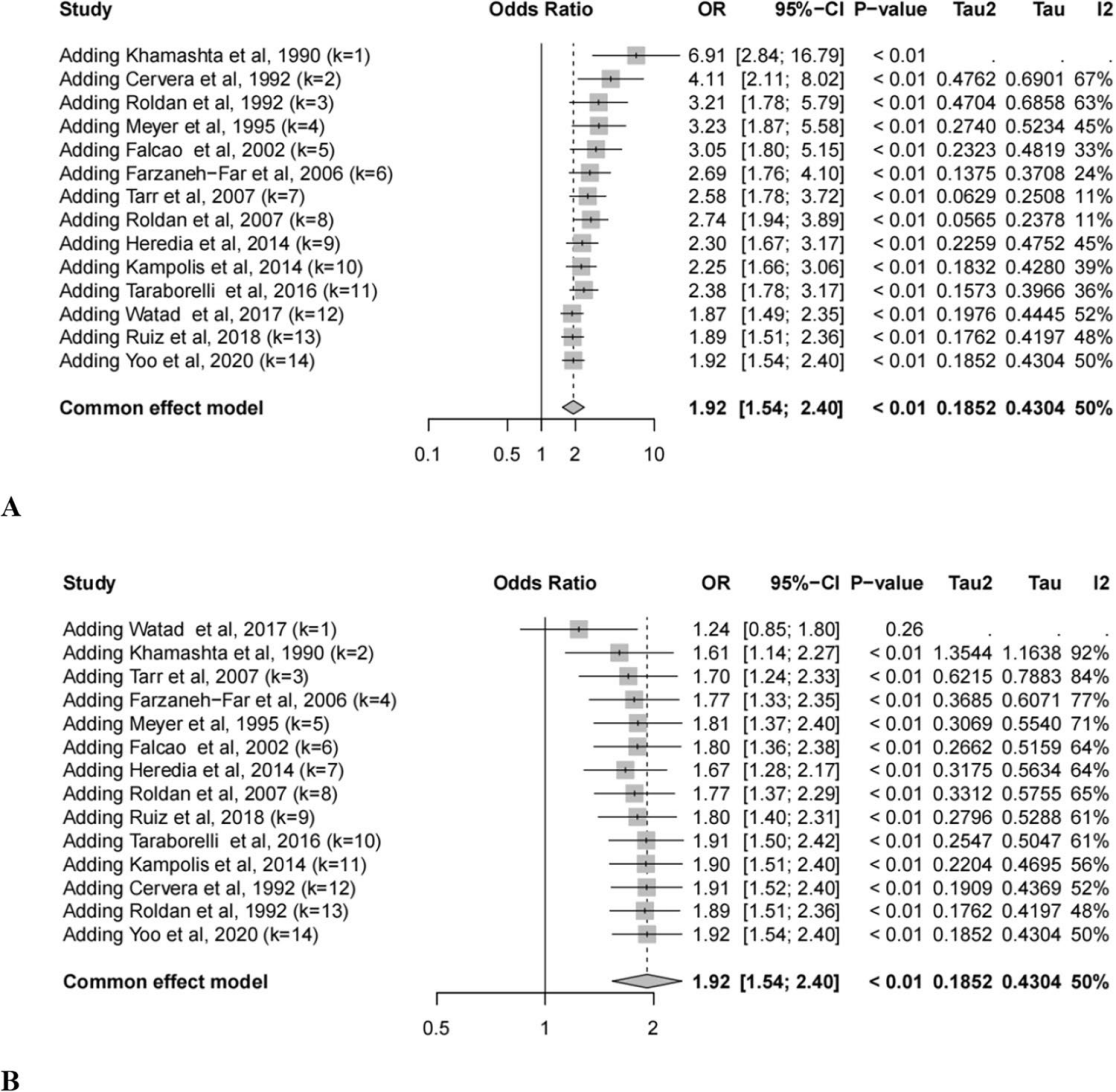
Cumulative meta-analyses of heart valve disease linked to antiphospholipid antibodies in SLE patients by publication date **A** and study size **B**

Our cumulative meta-analysis illustrated a time-dependent convergence of ORs toward a modest but statistically significant association of total aPLs. The earliest study conducted by Khamashta et al. in 1990 reported a substantial risk (OR = 6.91, 95% CI: 2.84–16.79), suggesting a strong association between aPLs and valvulopathy. As subsequent studies were included, there was an apparent attenuation of the ORs, which stabilized after the inclusion of Watad et al. (OR = 1.87, 95% CI: 1.49–2.35). The final pooled estimate yielded an OR of 1.92 (95% CI: 1.60–2.45, *p* < 0.01) with moderate heterogeneity (*I*^2^ = 50%, *τ*^2^ = 0.185), indicating a persistent and significant association (Fig. 4A). When ordered by increasing study size (Fig. 4B), as the cumulative meta-analysis progresses and larger studies are included, the OR was stabilized to around 2.

### Assessment of publication bias

**Fig. 5:**
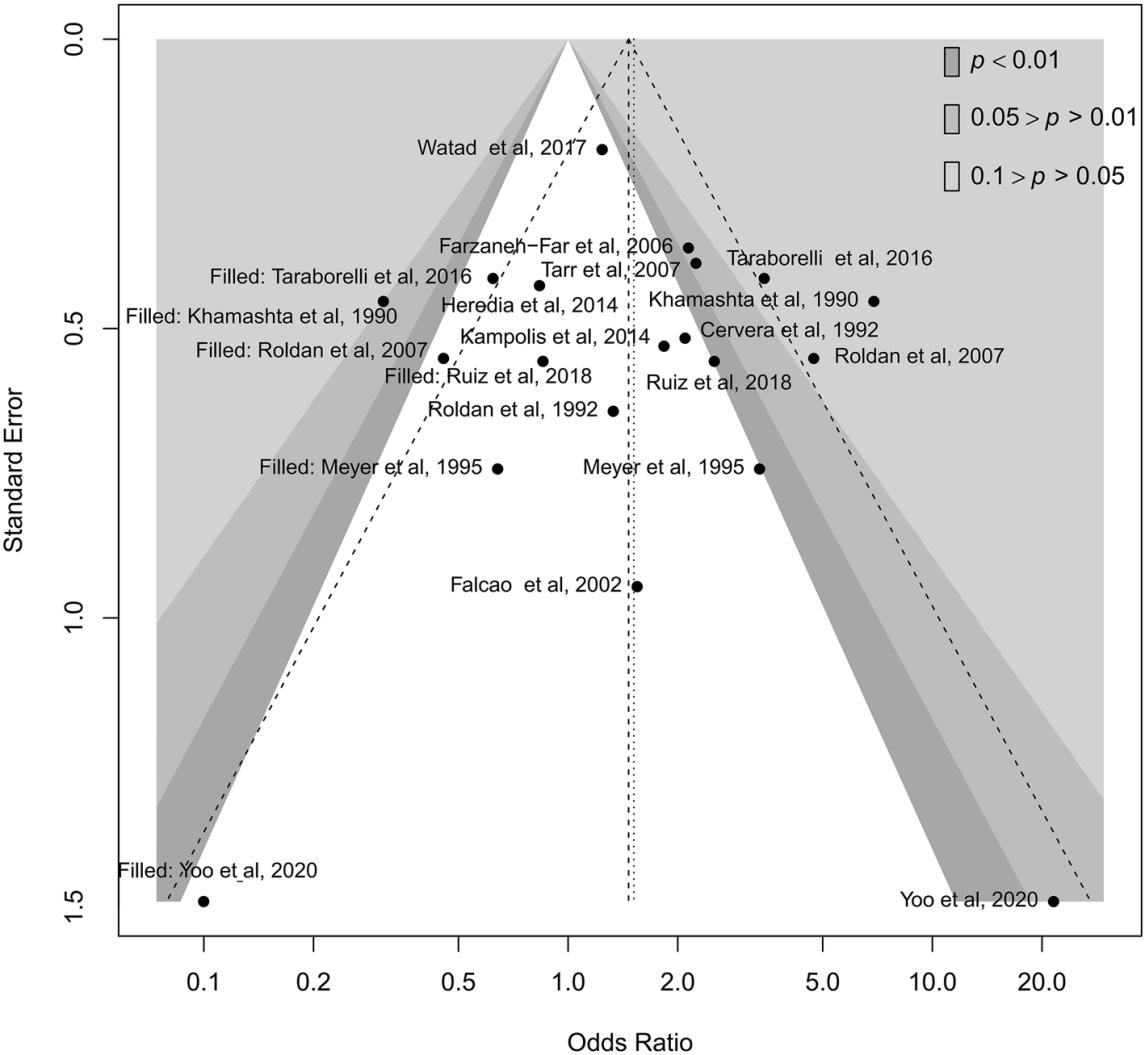
Enhanced contour funnel plot for trim-and-fill analysis of publication bias in aPL-positive patients with and without valvular lesions

Our evaluation of publication bias in the 14 studies examining the association between heart valve disease and total aPLs in SLE patients utilized Egger's test, which yielded a significant result (*p* = 0.046). This finding, coupled with the observed asymmetry in the corresponding funnel plot, suggested potential publication bias. The application of the trim-and-fill method, an approach to adjust for such bias, led to the inclusion of six additional hypothetical studies. This adjusted analysis, represented in the enhanced funnel plot (Fig. 5), placed five of these studies within the 95% CI and one outside, further indicating the likelihood of publication bias.

Despite these adjustments, the revised meta-analytic results continued to demonstrate a statistically significant association between aPL positivity and increased risk of heart valve disease in SLE patients. Specifically, the adjusted OR was 1.47 (95% CI: 1.20–1.79), indicating that aPLs are associated with a nearly 50% increase in the risk of heart valve disease.

## Discussion

Our meta-analysis of 25 studies offered a comprehensive assessment of the role of aPLs in heart valve disease among patients with SLE. This analysis is particularly critical in light of recent developments in the understanding of SLE and its cardiovascular implications. Heart valve disease in SLE is characterized by distinct pathological features, including vegetations on valve surfaces comprising various cellular components, leading to potential complications like thromboembolism or heart failure [2, 3]. Compared to previous meta-analyses on this topic, the present study has significantly increased the sample size [38, 39]. Additionally, the included studies demonstrate greater homogeneity in outcomes, and this represents the first meta-analysis to investigate the association between aβ2GPI and valvulopathy [30, 31].

Our findings revealed that the presence of aPLs nearly doubled the risk of heart valve disease in SLE patients. This significant association persists even after adjusting for potential publication bias, thereby reinforcing the contribution of aPLs to heart valve pathology in SLE, as aligned with previous studies [38, 39]. Notably, our study underscored the relevance of the new 2023 APS classification criteria [10], which includes cardiac valvular disease as a clinical criterion, primarily in patients with primary APS. Given the scarcity of research focusing on SLE patients in this context, our results suggested that these new criteria may be equally applicable in diagnosing APS among the SLE population.

Building further into the impact of specific aPL subtypes, we observed that aCL-IgG, aCL-IgM, and LA positivity were notably associated with increased risk of heart valve disease in SLE patients. These findings resonated with those of Zuily et al. and other researchers [39–42], highlighting a consistent trend across various studies. However, the meta-analyses conducted on aCL, aCL-IgG, and aCL-IgM yielded results that appear contradictory (Supplementary Fig. S3). Specifically, the ORs for aCL were significantly lower compared to the ORs for aCL-IgG and aCL-IgM. The primary reasons for these discrepancies likely stem from differences in study populations, antibody detection methods, and cutoff selection, which affect the interpretation of the positivity rates of different isotype antibodies under a uniform standard. The aCL group likely included a more heterogeneous population, and the studies for aCL often had significant temporal differences, incorporating more studies post-2010 compared to those for aCL-IgM and aCL-IgG. This heterogeneity and the inclusion of later studies might have diluted the apparent effect size for aCL compared to the more specific aCL-IgG and aCL-IgM groups. Additionally, significant differences in the positivity thresholds for aCL across studies, particularly in earlier research, contribute to the observed contradictions. These varying thresholds and methodological differences underscore the complexity and potential dilution effect in effect size, necessitating a more nuanced interpretation. The impact of anti-β2 glycoprotein I (aβ2GPI) seems relatively slight. The differential roles of these antibodies suggest diverse pathophysiological mechanisms, potentially involving endothelial damage, pro-inflammatory cytokines, and thrombotic processes. Understanding these mechanisms is imperative for a comprehensive view of heart valve disease in SLE [43–45].

Research suggests that various combinations of antiphospholipid antibodies may be associated with specific clinical phenotypes. For instance, triple positivity was potentially associated with microvascular events, including heart valve disease [46]. However, upon reviewing the included studies, we found that only a small portion provided the necessary information related to these associations. Consequently, conducting a meta-analysis on this aspect remains significantly limited at present. While longer disease duration and higher disease activity emerged as potential risk factors [5, 13, 18], heterogeneity in the study designs precluded a more thorough meta-analysis of these aspects. Treatment remains varied, with some reports suggesting benefits from immunosuppressive and antithrombotic therapies [13, 22, 47]. However, the high variability in treatment approaches necessitates more standardized and controlled studies to identify optimal management strategies.

Regarding valve-specific involvement, our analysis confirms the mitral valve as the most commonly affected, followed by aortic valve (Fig. 3), aligning with Vivero, Florencia et al.'s findings [6]. However, a meta-analysis by Hussain et al. and others indicated that the frequency of heart valve involvement in SLE patients, in descending order, is the mitral valve, tricuspid valve, aortic valve, and pulmonary valve [38]. Our study, which includes research containing data on aPLs, has differing inclusion criteria compared to that of Hussain et al. resulting in variations in the studies incorporated. The frequency of involvement for both the tricuspid and aortic valves in our included studies largely aligns with that reported by Hussain et al., with the exception of the studies by Higuera-Ortiz et al. [48] and Crozier et al. [49], where the incidence of tricuspid valve abnormalities is significantly higher than that of the aortic valve, marking it as the most affected valve. If these two studies are excluded, the incidence rates for tricuspid and aortic valve involvement are comparable. However, a comparison of patient characteristics between these two studies and others, particularly in terms of the proportion of pulmonary hypertension, does not confirm the reason for these discrepancies. Moreover, the distribution and types of lesions are critical for guiding patient stratification and enhancing the identification and management of aPL-related valvular diseases. Despite our attempts to conduct a meta-analysis to elucidate the risk of different valves involvement, the limited number of studies reduced the statistical significance of these associations (Supplementary Fig. S4). We anticipate that future research with more precise stratification will yield more robust data.

In summary, this meta-analysis underscores the critical role of aPLs in exacerbating heart valve disease in SLE patients. The differential impacts of different aPL subtypes on heart valve disease highlight the need for personalized clinical management, including regular cardiac assessments and tailored pharmacological interventions. Echocardiography proves to be an essential tool in this setting, enabling the early detection and ongoing monitoring of cardiac involvement in SLE patients. Although the current therapeutic experiences indicate potential benefits from certain medications, definitive treatment protocols for aPL-related HVD remain undefined. Consequently, we recommend routine echocardiographic screening for all SLE patients to promptly identify and manage cardiac complications, thereby potentially improving patient outcomes. Future research, especially large-scale randomized controlled trials, is crucial to establish evidence-based treatment guidelines and enhance patient care for SLE-related heart valve disease.

## Supplementary Information

Below is the link to the electronic supplementary material.

Supplementary file1 (DOCX 2187 kb)
