# Supplementary material for: Impact of antiphospholipid antibodies on cardiac valve lesions in systemic lupus erythematosus: a systematic review and meta-analysis
Source: Clin Exp Med. 2024 Jul 3;24(1):147. doi: 10.1007/s10238-024-01406-z (PMC11222203; doi:10.1007/s10238-024-01406-z)
Supplement: Supplementary file 1 — Supplementary file1 (DOCX 2187 kb) [file 10238_2024_1406_MOESM1_ESM.docx]

**Supplementary Table 1. Search Terms and Results for the Systematic Review**

| Source | Search terms | Filter | Results Yielded |
| --- | --- | --- | --- |
| Pubmed | ("Lupus Erythematosus, Systemic"[MeSH] OR "Systemic Lupus Erythematosus"[tiab] OR "Lupus Erythematosus Disseminatus"[tiab] OR "SLE"[tiab]) AND ("Antibodies, Antiphospholipid"[MeSH] OR "Antiphospholipid Autoantibody"[tiab] OR "Phospholipid Autoantibody"[tiab] OR "Antiphospholipid Antibody"[tiab] OR "Antibody, Antiphospholipid"[tiab] OR "Phospholipid Autoantibodies"[tiab] OR "Antiphospholipid Autoantibodies"[tiab] OR "Antiphospholipid Antibodies"[tiab] OR "aPLs"[tiab] OR "beta2GPI autoantibody"[tiab] OR "anti-beta2 GPI autoantibody"[tiab] OR "beta 2 glycoprotein I autoantibody"[tiab] OR "anti-beta2GPI autoantibody"[tiab] OR "anti-beta-2 glycoprotein I antibody"[tiab] OR "anti-β2 gpI"[tiab] OR "Antibodies, Anticardiolipin"[MeSH] OR "Anticardiolipin Autoantibody"[tiab] OR "Cardiolipin Autoantibodies"[tiab] OR "Anticardiolipin Antibody"[tiab] OR "Antibody, Anticardiolipin"[tiab] OR "Anticardiolipin Antibodies"[tiab] OR "Anticardiolipin Autoantibodies"[tiab] OR "Cardiolipin Autoantibody"[tiab] OR "ACL"[tiab] OR "Lupus Coagulation Inhibitor"[MeSH] OR "Lupus Anticoagulant Autoantibody"[tiab] OR "Anticoagulant, Lupus"[tiab] OR "Coagulation Inhibitor, Lupus"[tiab] OR "Lupus Anticoagulant Autoantibodies"[tiab] OR "Lupus Anticoagulant"[tiab] OR "LA"[tiab] OR "LAC"[tiab]) AND ("Libman-Sacks Disease"[tiab] OR "Disease, Libman-Sacks"[tiab] OR "Libman Sacks Disease"[tiab] OR "valve thickening"[tiab] OR "valve vegetation"[tiab] OR "cardic"[tiab] OR "valve"[tiab] OR "valvular lesion"[tiab] OR "valvulopathy"[tiab] OR "echocardiography"[tiab]) | English  Chinese | 202 |
| Web  of science | TS=(Lupus Erythematosus Systemic OR Systemic Lupus Erythematosus OR Lupus Erythematosus Disseminatus OR SLE) AND TS=(Antibodies Antiphospholipid OR Antiphospholipid Autoantibody OR Phospholipid Autoantibody OR Antiphospholipid Antibody OR Antibody Antiphospholipid OR Phospholipid Autoantibodies OR Antiphospholipid Autoantibodies OR Antiphospholipid Antibodies OR aPLs OR beta2GPI autoantibody OR anti-beta2 GPI autoantibody OR beta 2 glycoprotein I autoantibody OR anti-beta2GPI autoantibody OR anti-beta-2 glycoprotein I antibody OR anti-β2 gpI OR Antibodies Anticardiolipin OR Anticardiolipin Autoantibody OR Cardiolipin Autoantibodies OR Anticardiolipin Antibody OR Antibody Anticardiolipin OR Anticardiolipin Antibodies OR Anticardiolipin Autoantibodies OR Cardiolipin Autoantibody OR ACL OR Lupus Coagulation Inhibitor OR Lupus Anticoagulant Autoantibody OR Anticoagulant Lupus OR Coagulation Inhibitor Lupus OR Lupus Anticoagulant Autoantibodies OR Lupus Anticoagulant OR LAC OR LA) AND TS=(Libman-Sacks Disease OR Disease Libman-Sacks OR Libman Sacks Disease OR valve thickening OR valve vegetation OR cardic OR valve OR valvular lesion OR valvulopathy OR echocardiography) | English Chinese | 722 |
| embase | 'systemic lupus erythematosus'/syn AND ('phospholipid antibody'/syn OR 'antiphospholipid syndrome'/syn OR 'cardiolipin antibody'/syn OR 'lupus anticoagulant'/syn OR 'beta2 glycoprotein 1'/syn) AND ('valve'/syn OR 'valvulopathy'/syn OR 'echocardiography'/syn  ) NOT review | English Chinese | 915 |
| Cochrane | (Lupus Erythematosus, Systemic:ti,ab,kw OR Systemic Lupus Erythematosus:ti,ab,kw OR Lupus Erythematosus Disseminatus:ti,ab,kw OR SLE:ti,ab,kw) AND (Antibodies, Antiphospholipid:ti,ab,kw OR Antiphospholipid Autoantibody:ti,ab,kw OR Phospholipid Autoantibody:ti,ab,kw OR Antiphospholipid Antibody:ti,ab,kw OR Antibody, Antiphospholipid:ti,ab,kw OR Phospholipid Autoantibodies:ti,ab,kw OR Antiphospholipid Autoantibodies:ti,ab,kw OR Antiphospholipid Antibodies:ti,ab,kw OR aPLs:ti,ab,kw OR beta2GPI autoantibody:ti,ab,kw OR anti-beta2 GPI autoantibody:ti,ab,kw OR beta 2 glycoprotein I autoantibody:ti,ab,kw OR anti-beta2GPI autoantibody:ti,ab,kw OR anti-beta-2 glycoprotein I antibody:ti,ab,kw OR anti-β2 gpI:ti,ab,kw OR Antibodies, Anticardiolipin:ti,ab,kw OR Anticardiolipin Autoantibody:ti,ab,kw OR Cardiolipin Autoantibodies:ti,ab,kw OR Anticardiolipin Antibody:ti,ab,kw OR Antibody, Anticardiolipin:ti,ab,kw OR Anticardiolipin Antibodies:ti,ab,kw OR Anticardiolipin Autoantibodies:ti,ab,kw OR Cardiolipin Autoantibody:ti,ab,kw OR ACL:ti,ab,kw OR Lupus Coagulation Inhibitor:ti,ab,kw OR Lupus Anticoagulant Autoantibody:ti,ab,kw OR Anticoagulant, Lupus:ti,ab,kw OR Coagulation Inhibitor, Lupus:ti,ab,kw OR Lupus Anticoagulant Autoantibodies:ti,ab,kw OR Lupus Anticoagulant:ti,ab,kw OR LAC:ti,ab,kw OR LA:ti,ab,kw) AND (valve thickening:ti,ab,kw OR valve vegetation:ti,ab,kw OR Libman-Sacks Disease:ti,ab,kw OR Libman Sacks Disease:ti,ab,kw OR Disease,Libman-Sacks:ti,ab,kw OR valve:ti,ab,kw) |  | 0 |
| Total:1350 | | | |

| **Supplementary Table 2. Bias Rating Using the Newcastle-Ottawa Scale** | | | | | | | | |
| --- | --- | --- | --- | --- | --- | --- | --- | --- |
| **Study** | **Selection** | | | | **Comparability** | **Outcome/Exposure** | | |
| Cohort | Representativeness of the exposed cohort | Selection of the non exposed cohort | Ascertainment of exposure | Demonstration that outcome of interest was not present at start of study | Comparability of cohorts on the basis of the design or analysis | Assessment of outcome | Was follow-up long enough for outcomes to occur | Adequacy of follow up of cohorts |
| Nihoyannopoulos et al[17] | - | * | * | - | * | * | * | * |
| Khamashta et al[18]] | * | * | * | - | ** | * | - | * |
| Sturfelt et al[19] | * | * | * | - | ** | * | * | * |
| Cervera et al[20] | * | * | * | - | ** | * | - | * |
| Jouhikainen et al[21] | * | * | * |  | ** | * | * | * |
| Moyssakis et al[3] | * | * | * |  | ** | * | - | * |
| Kampolis et al[22] | * | * | * |  | ** | * | * | * |
| Case control | Is the case definition adequate? | Representativeness of the cases | Selection of Controls | Definition of Controls | Comparability of cases and controls on the basis of the design or analysis | Ascertainment of exposure | Same method of ascertainment for cases and controls | Non-Response rate |
| Ruid et al[14] | * | - | - | * | ** | * | * | - |
| Yoo et al[13] | * | - | * | * | ** | * | * | * |
| He et al[23] | * | - | * | * | ** | * | * | * |

| **Supplementary Table 3. Bias Rating Using the Agency for Healthcare Research and Quality (AHRQ) Scale** | | | | | | | | | | | |
| --- | --- | --- | --- | --- | --- | --- | --- | --- | --- | --- | --- |
| Study | Define source of information | List inclusion and exdusion criteria for exposed and unexposed subjects or refer to previous publications | Indicate time period used for identifying patients | Indicate whether or not subiects were consecutive if not population -based | Indicate if evaluators of subjective components of study were masked to other aspects of the status of the participants | Describe any assessments undertaken for quality assurance purposes | Explain any patient exclusions from analysis | Describe how confounding was assessed and/or controlled | lf applicable, explain how missing data were handled in the analysis | Summarize patient response rates and completeness of data collection | Clarify what follow-up, if any, was expected and the percentage of patients for which incomplete data or follow-up was obtained |
| Roldan et al[24] | * | * | - | - | * | * | * | * | - | * | * |
| Meyer et al[25] | * | * | * | * | * | * | * | - | - | * | * |
| Gabrielli et al[26] | * | * | - | - | * | * | * | - | * | * | * |
| Bengtsson et al[27] | * | * | * | - | - | - | - | - | * | * | * |
| Falcao et al[28] | * | * | - | * | * | - | * | * | * | * | * |
| Leszczynski et al[29] | * | * | - | - | - | * | * | * | * | * | * |
| Shahin et al[30] | * | * | - | - | * | - | * | * | * | * | * |
| Roldan et al[31] | * | * | - | - | * | - | * | - | * | * | * |
| Farzaneh-Far et al[32] | * | * | * | * | * | * | * | - | - | * | * |
| Roldan et al[33] | * | * | - | - | * | - | * | - | * | * | * |
| Tarr et al[34] | * | * | * | - | * | - | * | - | * | * | - |
| Heredia et al[35] | * | * | * | - | - | - | * | - | * | * | * |
| Taraborelli et al[15] | * | * | * | - | * | - | * | - | * | * | * |
| Watad et al[36] | * | - | * | - | * | - | - | - | * | * | * |
| Liao et al[37] | * | - | * | - | * | * | * | - | * | * | * |
| *: Yes; -: No or Unclear | | | | | | | | | | | |

**Supplementary Figure 1. Heterogeneity Analysis based on Baujat and Galbraith Radial Plots of Studies on aPL-Positive Patients With and Without Valvular Lesions**: **A. Galbraith radial plot of total aPLs; B. Baujat plot of total aPLs; C. Galbraith radial plot of aCL; D. Baujat plot of aCL; E. Galbraith radial plot of LA; F.Baujat plot of LA. aPLs: antiphospholipid antibodies; aCL: anticardiolipin antibody; aβ2GPⅠ: anti-β2 glycoprotein Ⅰ antibody; LA: lupus anticoagulant.**

| 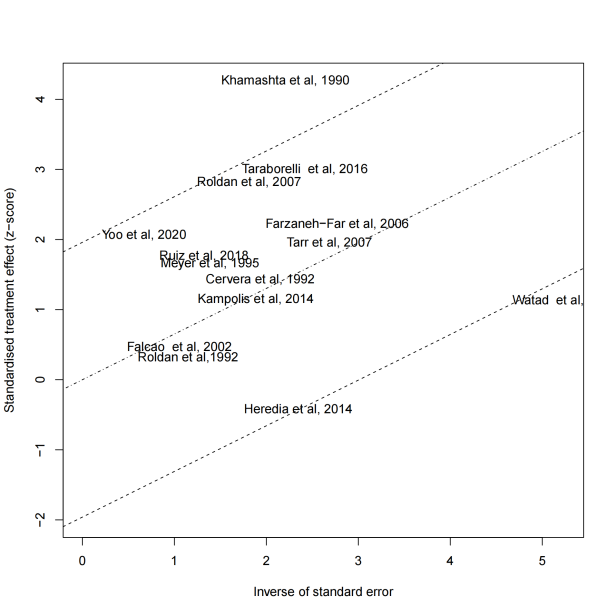 | 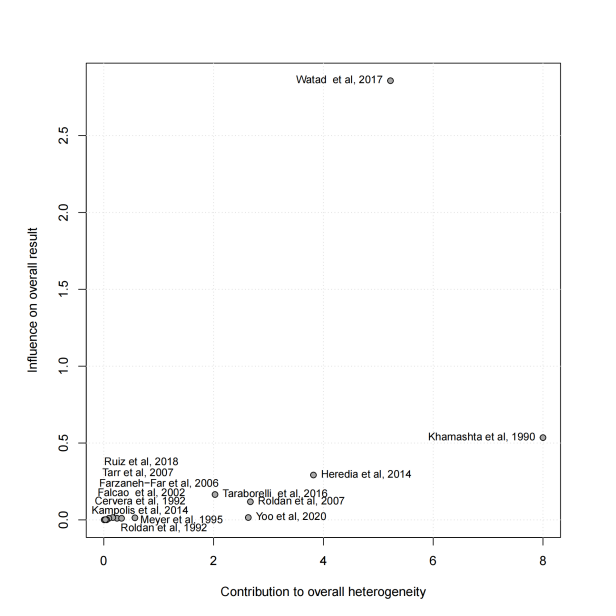 |
| --- | --- |
| A | B |
| 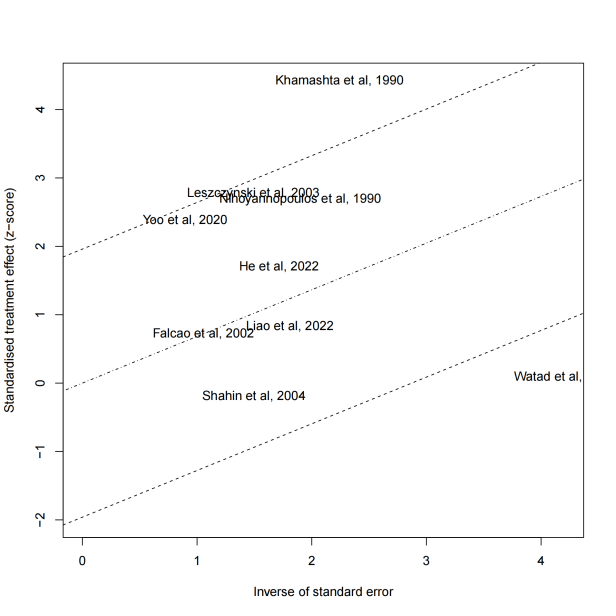 | 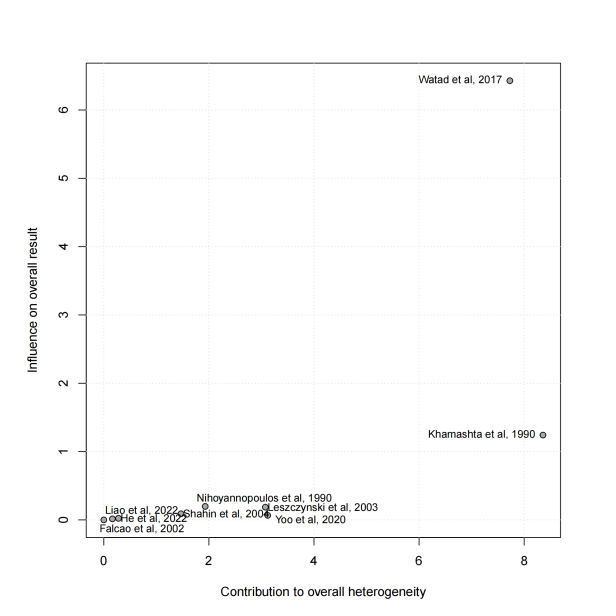 |
| C | D |
| 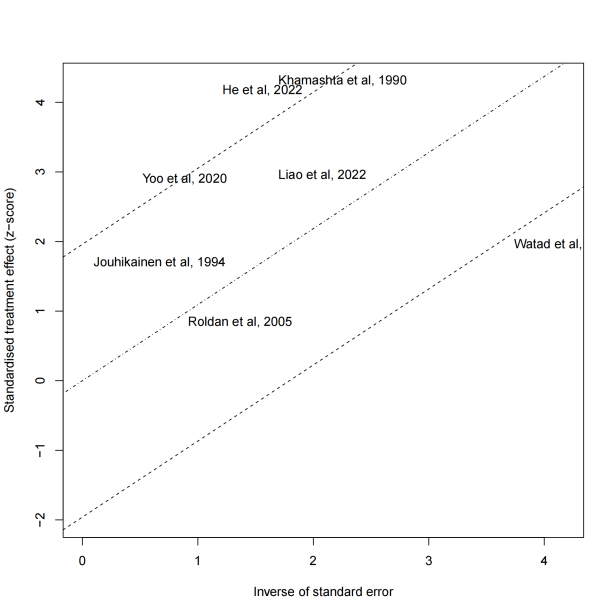 | 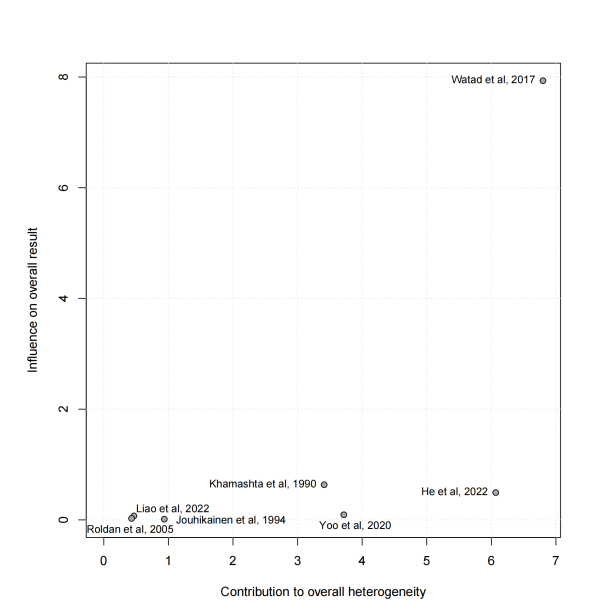 |
| E | F |

| **Supplementary Figure 2. Sensitivity Analysis: A. Total aPLs; B. aCL; C: aβ2GPⅠ; D. LA. aPLs: antiphospholipid antibodies; aCL: anticardiolipin antibody; aβ2GPⅠ: anti-β2 glycoprotein Ⅰ antibody; LA: lupus anticoagulant.** |
| --- |
| 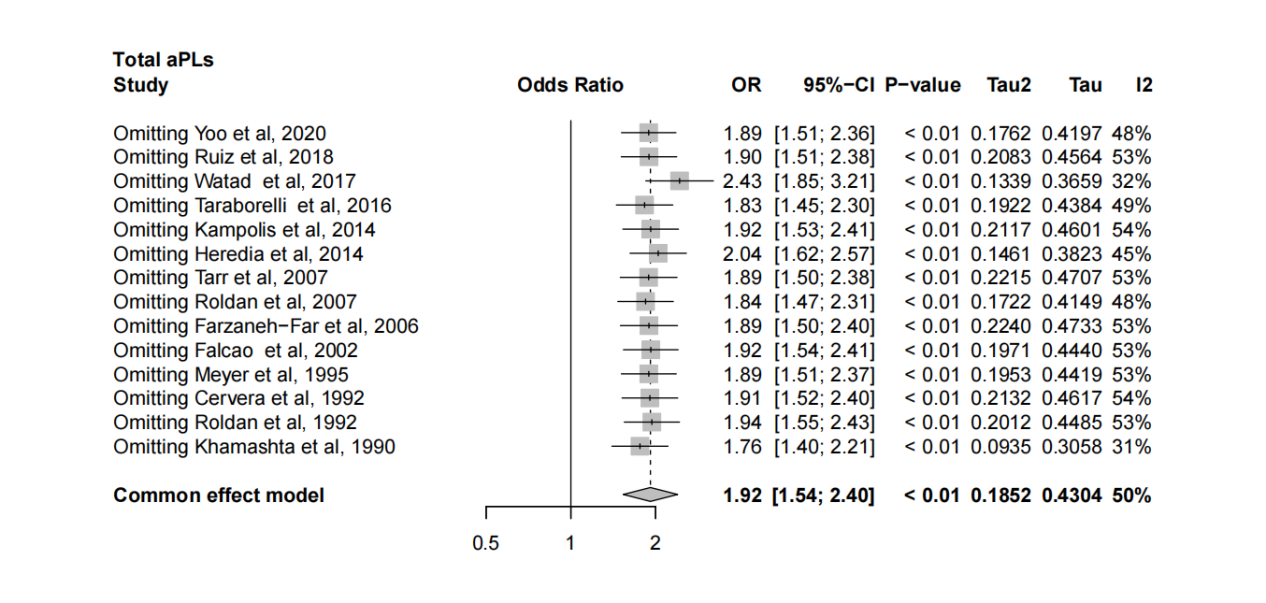   \| A \| \| --- \| \| 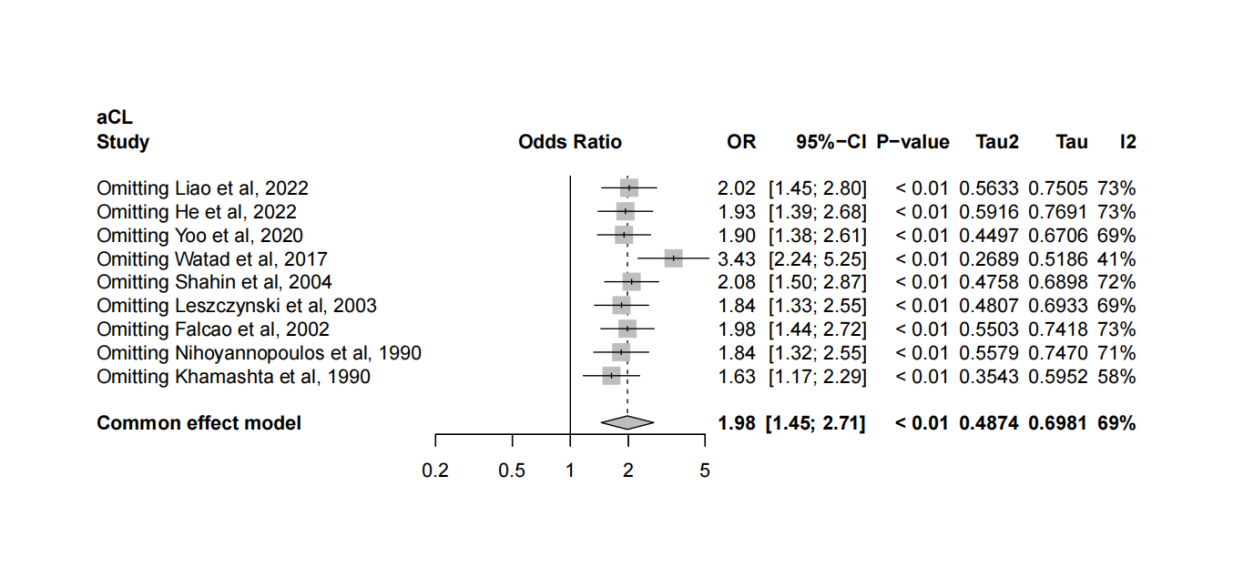 \| \| B \| \| 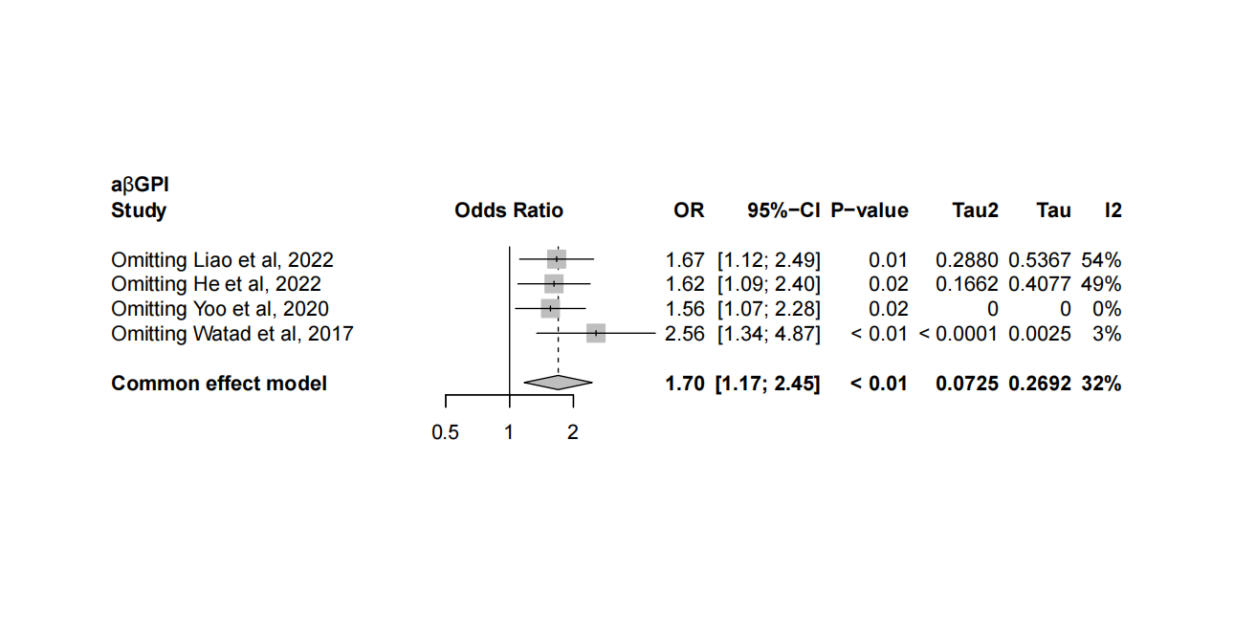 \| \| C \| \| 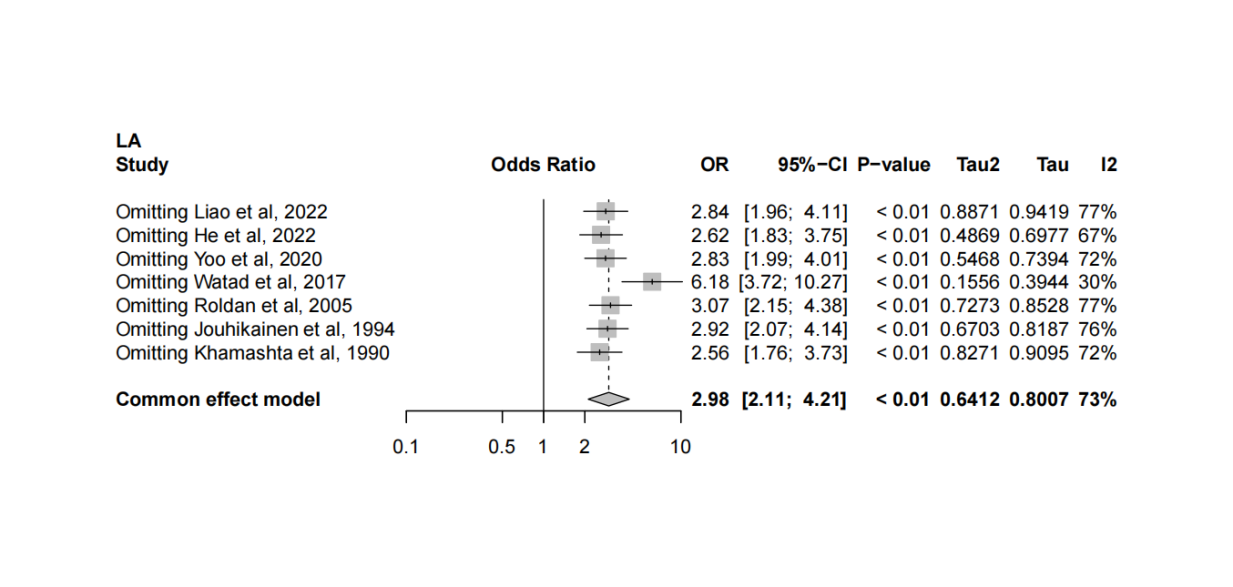 \| \| D \| |

**Supplementary Figure 3. Risk of Heart Valve Disease in patients with aCL-IgG or aCL-IgM**: **A. aCL-IgG; B. aCL-IgM. aCL: anticardiolipin antibody; HVD: heart vavle disease.**

| 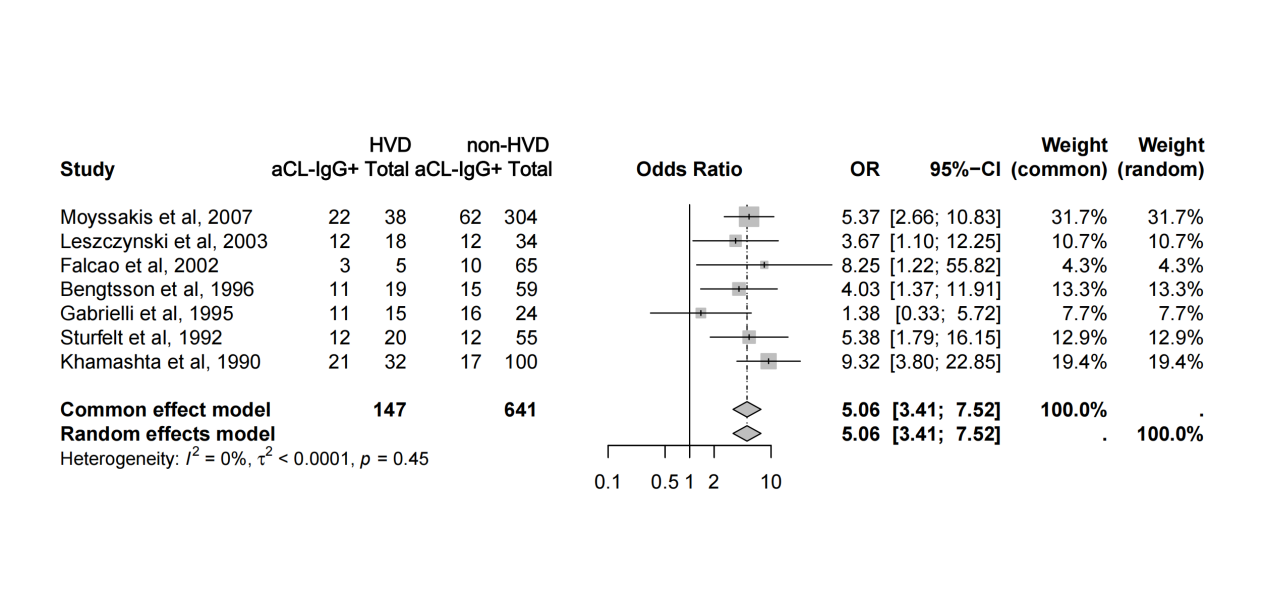 |
| --- |
| A |
| 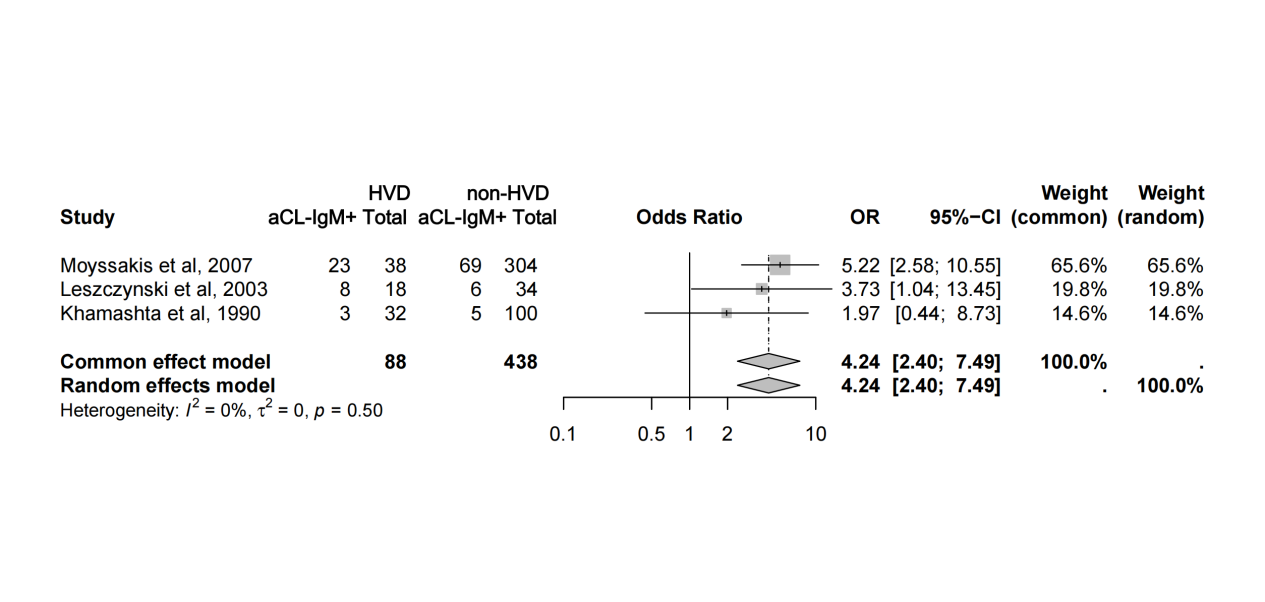 |
| B |

**Supplementary Figure 4. Risk of Various Heart Valve Disease Associated with Antiphospholipid Antibodies in SLE: A. Mitral valve; B. Tricuspid valve; C. Aortic valve; D. Pulmonary valve. aPLs: antiphospholipid antibodies.**

| 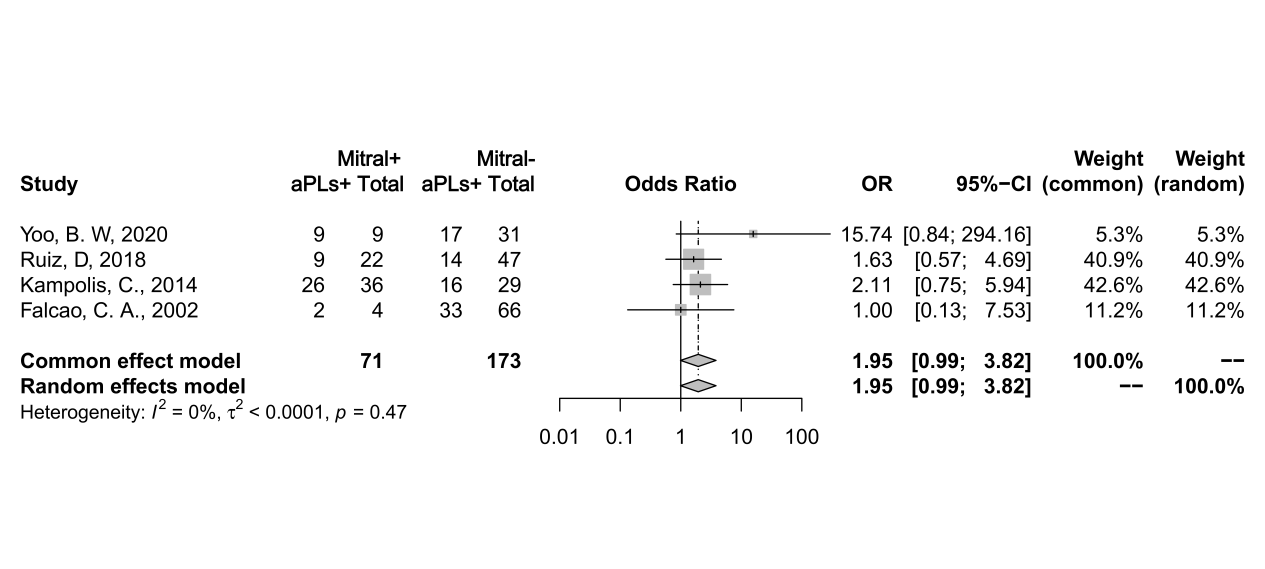 |
| --- |
| A |
| 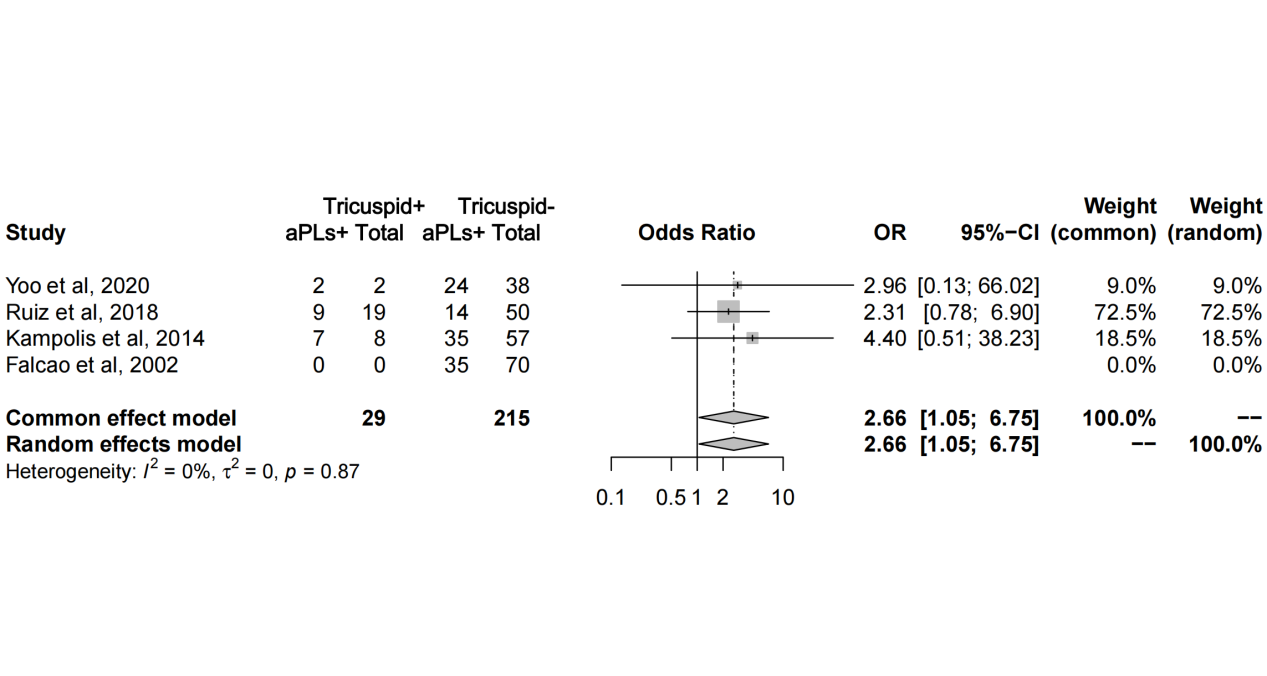 |
| B |
| 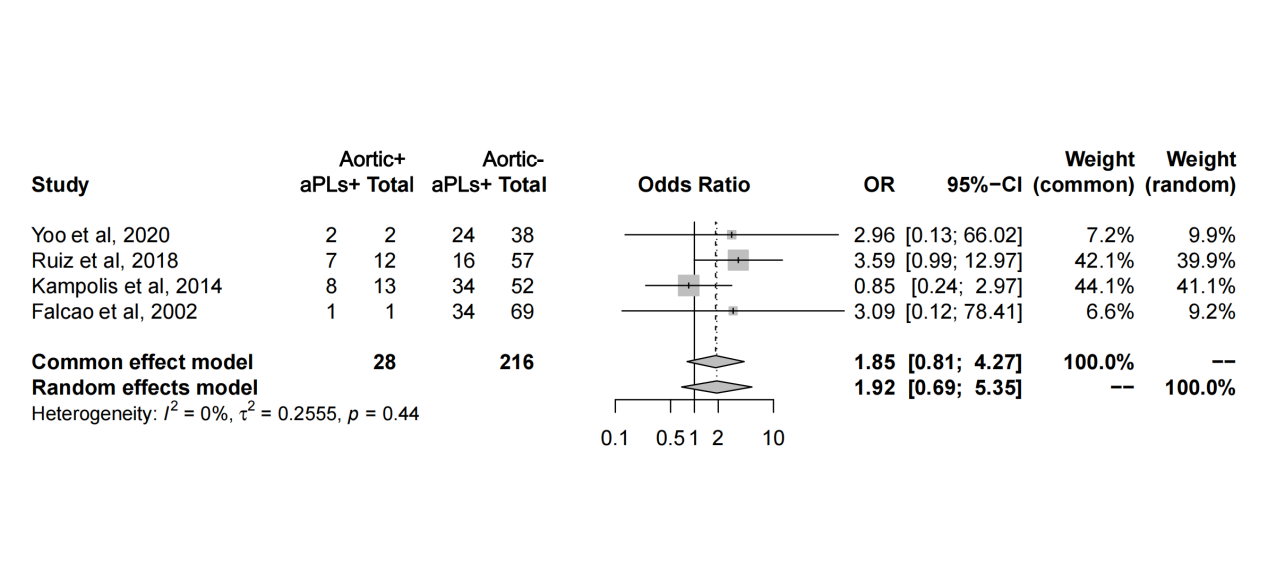 |
| C |
| 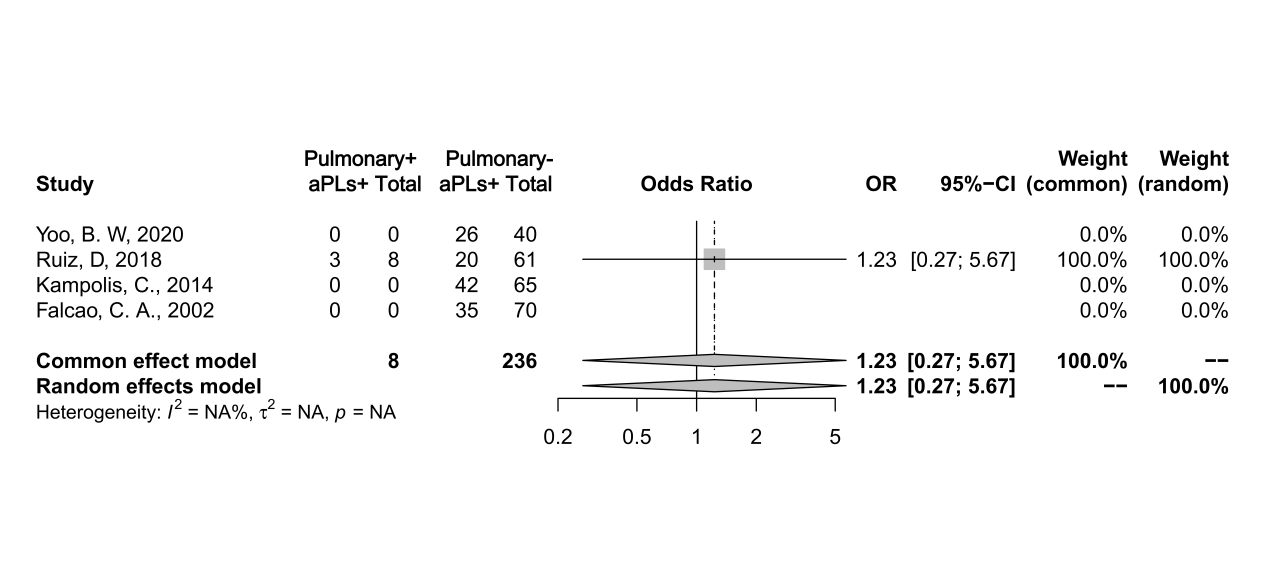 |
| D |
